# Sex- and reproductive status-specific relationships between body composition and non-alcoholic fatty liver disease

**DOI:** 10.1186/s12876-023-02997-9

**Published:** 2023-10-24

**Authors:** Yu-tian Cao, Wen-hui Zhang, Yan Lou, Qian-hua Yan, Yu-juan Zhang, Fang Qi, Liu-lan Xiang, Tian-su Lv, Zhu-yuan Fang, Jiang-yi Yu, Xi-qiao Zhou

**Affiliations:** 1 Department of Endocrinology, Affiliated Hospital of Nanjing, Jiangsu Province Hospital of Chinese Medicine, University of Chinese Medicine, Nanjing, China; 2 The First School of Clinical Medicine, Nanjing University of Chinese Medicine, Nanjing, China; 3 Institute of Hypertension, Jiangsu Province Hospital of Chinese Medicine, Affiliated Hospital of Nanjing University of Chinese Medicine, Nanjing, China

**Keywords:** Sex, Menopause, Body composition, Muscle, Fat, NAFLD

## Abstract

**Background:**

Sex and reproductive status differences exist in both non-alcoholic fatty liver disease (NAFLD) and body composition. Our purpose was to investigate the relationship between body composition and the severity of liver steatosis and fibrosis in NAFLD in different sex and reproductive status populations.

**Methods:**

This cross-sectional study included 880 patients (355 men, 417 pre-menopausal women, 108 post-menopausal women). Liver steatosis and fibrosis and body composition data were measured using FibroScan and a bioelectrical impedance body composition analyzer (BIA), respectively, and the following parameters were obtained: liver stiffness measurement (LSM), controlled attenuation parameter (CAP), waist circumference (WC), body mass index (BMI), percent body fat (PBF), visceral fat area (VFA), appendicular skeletal muscle mass (ASM), appendicular skeletal muscle mass index (ASMI), fat mass (FM), fat free mass (FFM), and FFM to FM ratio (FFM/FM). Multiple ordinal logistic regression (MOLR) was used to analyze the independent correlation between body composition indicators and liver steatosis grade and fibrosis stage in different sex and menopausal status populations.

**Results:**

Men had higher WC, ASM, ASMI, FFM, and FFM/FM than pre- or post-menopausal women, while pre-menopausal women had higher PBF, VFA, and FM than the other two groups (p < 0.001). Besides, men had greater CAP and LSM values (p < 0.001). For MOLR, after adjusting for confounding factors, WC (OR, 1.07; 95% CI, 1.02–1.12; P = 0.011) and FFM/FM (OR, 0.52; 95% CI, 0.31–0.89; P = 0.017) in men and visceral obesity (OR, 4.16; 95% CI, 1.09–15.90; P = 0.037) in post-menopausal women were independently associated with liver steatosis grade. WC and visceral obesity were independently associated with liver fibrosis stage in men (OR, 1.05; 95% CI, 1.01–1.09, P = 0.013; OR, 3.92; 95% CI, 1.97–7.81; P < 0.001, respectively).

**Conclusions:**

Increased WC and low FFM/FM in men and visceral obesity in post-menopausal women were independent correlates of more severe liver steatosis. In addition, increased WC and visceral obesity were independent correlates of worse liver fibrosis in men. These data support the sex- and reproductive status-specific management of NAFLD.

**Supplementary Information:**

The online version contains supplementary material available at 10.1186/s12876-023-02997-9.

## Introduction

Non-alcoholic fatty liver disease (NAFLD) is the most common liver disease, with a worldwide prevalence of 25.24% [1, 2]. The broader term “NAFLD” encompasses the full spectrum of fatty liver disease, from simple hepatic steatosis or nonalcoholic fatty liver to nonalcoholic steatohepatitis (NASH) and NASH cirrhosis [3]. The incidence and prevalence of NAFLD have increased dramatically in recent years, paralleling the global epidemics of obesity and diabetes mellitus, which have also placed a huge burden on public health [1].

Sex differences are common in diseases, including NAFLD. This is reflected in the prevalence, risk factors, fibrosis, and clinical outcomes of NAFLD [4]. The studies have found that the prevalence of NAFLD is lower in women of reproductive age, but it begins to rise in post-menopausal women, approaching or even exceeding the prevalence of NAFLD in men [5]. Important sex differences in body data, such as body fat and fat distribution, are also hidden when using the body mass index (BMI) alone as a measure of the human body [6]. Furthermore, certain body compositions, such as high visceral fat area (VFA) and low appendicular skeletal muscle mass (ASM), are also linked to NAFLD progression [7–9]. Notably, the same body data affects NAFLD differently in different sex populations. For example, one study found that waist circumference (WC) was associated with hepatic fat accumulation in men but not in women [10]. Therefore, NAFLD, sex and reproductive status, and body composition are all interrelated and influenced each other. However, there are few relevant studies. Taking sex, menopausal status, and physical data into account in NAFLD clinical studies can further refine NAFLD management.

The goal of this study was to explore how different sexes and menopausal status affect the relationship between body composition and the severity of steatosis and fibrosis in NAFLD.

## Patients and methods

### Study design and population

This is a cross-sectional study designed to investigate the relationship between body composition and the severity of steatosis and fibrosis in NAFLD in individuals of different sexes and menopausal status. The Strengthening the Reporting of Observational Studies in Epidemiology Statement has been followed during this study (Additional file: Table S1).

**Fig. 1:**
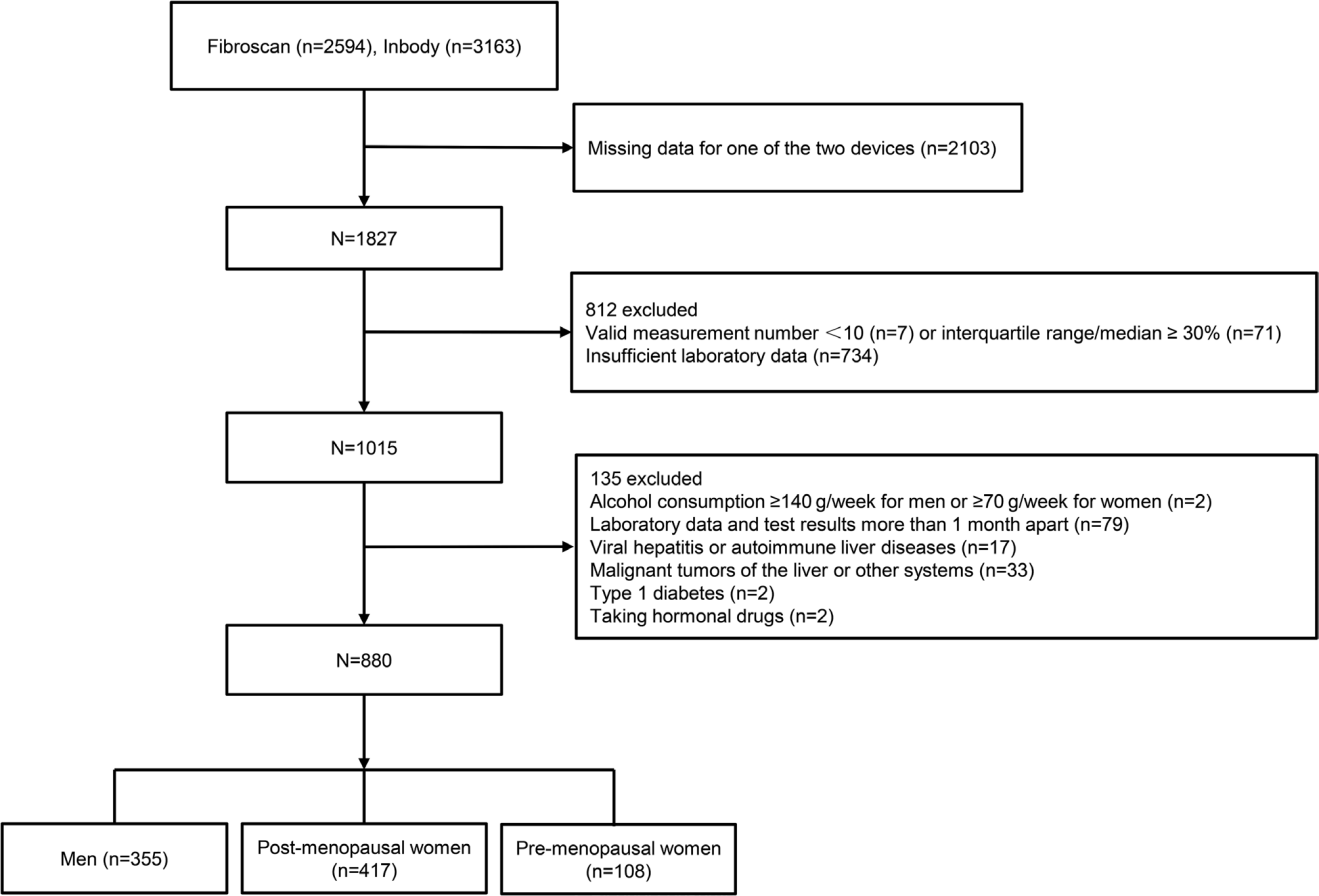
The flow chart of the inclusion and exclusion of participants in the study

We initially retrieved 2594 and 3163 records from the database that came with the FibroScan and Inbody devices, respectively, from patients who attended the Endocrinology Department of Jiangsu Provincial Hospital of Chinese Medicine between January 2022 and October 2022. Then, we screened out 1827 patients with both examination data (two examinations were performed on the same day). We then excluded 812 records (78 invalid test results; 734 insufficient laboratory data). We further excluded the following patients by reviewing their electronic medical records: with alcohol consumption ≥ 140 g/week for men or ≥ 70 g/week for women (n = 2); laboratory data and test results more than 1 month apart (n = 79); with viral hepatitis or autoimmune liver diseases (n = 17); with malignant tumors of the liver or other systems (n = 33); with type 1 diabetes (n = 2); taking hormonal drugs (n = 2). Finally, 880 patients were included in this study (355 men; 417 pre-menopausal women; 108 post-menopausal women) (Fig. 1).

The study complied with both the Declarations of Helsinki and Istanbul for the participation of human subjects in research and was approved by the Ethics Committees of Jiangsu Provincial Hospital of Chinese Medicine, Affiliated Hospital of Nanjing University of Chinese Medicine (2022NL-071-02). In this retrospective study, we collected data by clinic ID number rather than name to protect patient privacy. Therefore, informed consent was waived for this study.

### Liver steatosis and fibrosis measurement

Liver stiffness measurement (LSM) and controlled attenuation parameter (CAP) were obtained by professional technicians using transient elastography—FibroScan equipment (Echosens, Paris, France), who were blind to clinical data. Probe selection was based on BMI and an automated probe selection tool [11]. The measurement results were considered reliable when there were ≥ 10 successful measurements and the interquartile range (IQR)/median was < 30%, and the success rate was ≥ 60% [12].

Liver steatosis and fibrosis grading were classified using previously described liver biopsy-support thresholds derived from a meta-analysis in NAFLD patients [13]. The diagnostic values of S1, S2, and S3 were 269, 288, and 313 dB/m, respectively. For the division of liver fibrosis from F1-F4, the thresholds were 6.7, 7.6, 9.8, and 12.9 kPa, respectively.

### Body composition measurement

On the same day as the FibroScan test, body composition data was measured using a bioelectrical-impedance body composition analyzer (BIA) (InBody 770, Seoul, South Korea), and technicians were blinded to the clinical information. Height, weight, WC, BMI, percent body fat (PBF), VFA, ASM, fat mass (FM), fat free mass (FFM), FFM to FM ratio (FFM/FM), and appendicular skeletal muscle mass index (ASMI) were the body data examined in this study. ASMI was corrected for height (ASMI = ASM (kg)/height (m²), kg/m²). According to the Asian Working Group for Sarcopenia (AWGS) criteria, sarcopenia is defined as ASMI < 7.0 kg/ m² in men and < 5.7 kg/ m² in women (measured by BIA) [14]. Visceral obesity was defined as VFA ≥ 100 cm² in both sexes [15].

### Collection of other clinical data

Electronic medical records of patients were reviewed retrospectively to collect demographic and other clinical data. For menopause, if no records were available, it was divided according to the age of 49 (the average menopausal age of Chinese women) [16]. All blood samples from patients undergoing biochemical analysis were obtained after at least an 8-hour overnight fast, and the testers were also blinded to clinical data. Only laboratory data obtained within 1 month of the FibroScan test were considered valid (multiple data, whichever was closest), including serum levels of alanine aminotransferase (ALT), aspartate aminotransferase (AST), γ­-glutamyl transferase (GGT), alkaline phosphatase (AKP), fasting blood glucose (FBG), fasting insulin (FINS), homeostasis model assessment of insulin resistance (HOMA-IR), total cholesterol (TC), triglyceride (TG), high-density lipoprotein (HDL), and low-density lipoprotein (LDL). HOMA-IR = FINS (µIU/mL) ×FBG (mmol/L)/22.5 was used to assess insulin resistance (IR) [17].

Type 2 diabetes mellitus (T2DM) was defined as FBG ≥ 7.0 mmol/L or 2-h plasma glucose ≥ 11.1 mmol/L or hemoglobin A1c (HbA1c) ≥ 6.5%, and/or treatment with anti-diabetic medication currently [18]. The definition of dyslipidemia was TG ≥ 2.3 mmol/L or TC ≥ 6.2 mmol/L or HDL < 1.0 mmol/L or LDL ≥ 4.1 mmol/L, and/or currently taking antihyperlipidemic medication [19].

### Statistical analyses

Continuous variables were presented as medians and IQRs, while categorical variables were presented as frequencies and percentages. Violin plots depict the distribution of continuous variables, whereas stacked histograms show the distribution of categorical variables. Analysis of variance (ANOVA) was used to compare data between three groups for continuous variables, and χ² test was used for categorical variables. Kruskal-Wallis (K-H) test was used to compare ordinal data between three groups. Post hoc analyses of the ANOVA, χ² test, and K-H test were performed using the Bonferroni method, partitions of χ² method, and Kwallis2 Stata module [20], respectively. Heat plots were used to visually inspect the associations between body composition data and CAP and LSM values.

Besides, we tested for interactions between (1) sex and body composition and (2) menopausal status and body composition. Subsequently, ordinal logistic regressions were performed with sex and menopause as stratifying factors for sex- and reproductive status-specific correlations between body composition indicators (as exposure factors) and steatosis grade and fibrosis stage (as outcomes). Furthermore, in order to explore whether these body factors are independently related to steatosis grade and fibrosis stage, we performed multivariate ordinal logistic regression (MOLR) and used three models: model 1, adjusted for age and BMI; model 2, adjusted for liver enzymes and dyslipidemia based on model 1; model 3, adjusted for T2DM and HOMA-IR based on model 2. All of the above models were tested for co-linearity, and co-linear variables were removed to improve the accuracy of the model parameter estimates. The odds ratio (OR) and 95% confidence interval (CI) were calculated to determine the significance of the association.

For multiple testing of post hoc analyses, corrected significance levels of p < 0.0167 (0.05/3) were used. For other analyses, statistical significance was defined as two-sided p < 0.05. All statistical tests and graphic creation were performed with Stata Version 15.0 (StataCorp).

## Result

### Patients’ characteristics

**Fig. 2:**
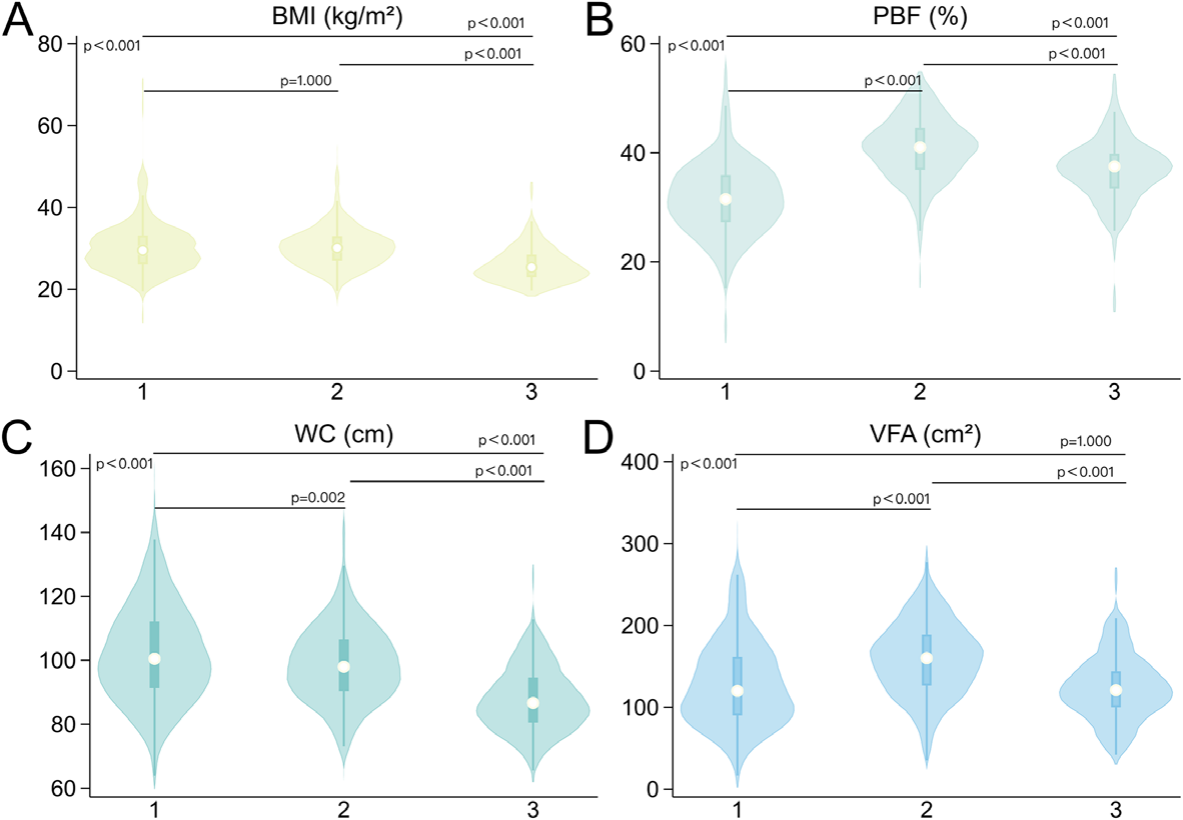
Comparison of BMI, PBF, WC, and VFA values between the three groups. 1. Men; 2. Pre-menopausal women; 3. Post-menopausal women; BMI: body mass index; PBF: percent body fat; WC: waist circumference; VFA: visceral fat area

This study included 880 patients, including 355 men, 417 pre-menopausal women, and 108 post-menopausal women. Approximately 80% of female patients reported reproductive status information. The average age of menopause among the female patients with records was 48.2 ± 3.3 years. Table 1 summarizes the clinical characteristics of patients stratified by sex and menopause. In terms of physical data, there were significant differences between the three groups. Men had higher WC, ASM, ASMI, FFM, and FFM/FM than pre- and post-menopausal women, while pre-menopausal women had higher PBF, VFA, and FM than the other two groups (p < 0.001, Fig. 2B-D A-D). Post hoc analyses showed similar BMI in men and pre-menopausal women (p = 1.000, Fig. 2A) and similar VFA in post-menopausal women and in men (p = 1.000, Fig. 2D). For the laboratory data, there were significant differences between the three groups (p < 0.001), except for HOMA-IR, TC, and LDL. Finally, regarding hepatological characteristics, both CAP and LSM values were significantly higher in men than in women (p < 0.001, Fig. 4A, C), while there were no significant differences in LSM values between pre- and post-menopausal women (p = 0.953, Fig. 4C). The distribution of liver steatosis and fibrosis grades was significantly different between the three groups (p < 0.001). Severe liver steatosis (S = S3) and advanced liver fibrosis (F ≥ F3) were more prevalent in men, followed by pre-menopausal women, and finally post-menopausal women (Fig. 4B, D). The test power was shown in Additional Table S2.

**Table 1:** Clinical characteristics of patients stratified by sex and menopause

	1. Men (n = 355)	2. Pre-menopausal women (n = 417)	3. Post-menopausal women (n = 108)	P value^a^	Post-hoc^b^		
					1 vs. 2	1 vs. 3	2 vs. 3
Demographic parameter							
Age (year)	39 (31, 49)	33 (28, 38)	57 (53, 63)	<0.001	<0.001	<0.001	<0.001
Body parameters							
BMI (kg/m^2^)	29.5 (26.1, 33.1)	30.1 (27.0, 32.9)	25.4 (22.95, 28.45)	<0.001	1.000	<0.001	<0.001
WC (cm)	100.5 (91.4, 112.1)	98.0 (90.4, 106.5)	86.7 (80.55, 94.55)	<0.001	0.002	<0.001	<0.001
PBF (%)	31.5 (27.3, 35.9)	41.0 (36.9, 44.5)	37.5 (33.5, 39.8)	<0.001	<0.001	<0.001	<0.001
VFA (cm^2^)	120.0 (90.4, 161.5)	160.1 (126.8, 188.7)	121.0 (100.1, 143.9)	<0.001	<0.001	1.000	<0.001
ASM (kg)	33.3 (29.8, 37.5)	25.2 (22.8, 27.7)	21.2 (19.3, 23.3)	<0.001	<0.001	<0.001	<0.001
ASMI (kg/m²)	8.6 (7.9, 9.3)	7.3 (6.8, 7.9)	6.4 (5.9, 6.9)	<0.001	<0.001	<0.001	<0.001
FM (kg)	27.6 (20.7, 35.4)	31.9 (26.0, 37.9)	22.9 (19.3, 27.7)	<0.001	<0.001	<0.001	<0.001
FFM (kg)	59.1 (53.2, 66.5)	45.8 (41.8, 50.2)	39.5 (36.2, 43.0)	<0.001	<0.001	<0.001	<0.001
FFM/FM	2.18 (1.79, 2.66)	1.44 (1.25, 1.71)	1.66 (1.51, 1.99)	<0.001	<0.001	<0.001	0.004
Biochemical parameters							
ALT (U/L)	36 (22, 61)	24 (15, 41)	20 (15, 32)	<0.001	<0.001	<0.001	0.024
AST (U/L)	23 (18, 35)	19 (15, 26)	20 (17, 26)	0.001	0.016	0.005	0.516
AKP (U/L)	80 (67, 99)	75 (62, 88)	86 (76, 101)	<0.001	<0.001	0.101	<0.001
GGT (U/L)	41 (25, 66)	26 (17, 42)	21 (16, 35)	<0.001	<0.001	<0.001	1.000
FBG (mmol/L)	5.89 (5.52, 7.41)	5.30 (4.95, 5.85)	6.68 (5.68, 8.03)	<0.001	<0.001	0.365	<0.001
FINS (µIU/mL)	10.28 (6.57, 16.69)	12.18 (7.89, 18.84)	7.02 (4.59, 10.75)	<0.001	0.592	<0.001	<0.001
HOMA-IR	2.94 (1.85, 4.65)	2.95 (1.90, 4.84)	1.98 (1.34, 3.21)	0.062			
TG (mmol/L)	2.01 (1.30, 2.91)	1.36 (0.98, 2.00)	1.54 (1.08, 2.09)	<0.001	<0.001	<0.001	1.000
TC (mmol/L)	4.99 (4.42, 5.71)	4.93 (4.43, 5.58)	5.37 (4.36, 5.90)	0.236			
HDL (mmol/L)	1.23 (1.08, 1.42)	1.39 (1.22, 1.54)	1.49 (1.34, 1.74)	<0.001	<0.001	<0.001	<0.001
LDL (mmol/L)	3.10 (2.62, 3.64)	3.01 (2.54, 3.49)	3.04 (2.37, 3.66)	0.172			
Concomitant diseases							
T2DM	239 (67.3)	156 (37.4)	92 (85.2)	<0.001	<0.001	<0.001	<0.001
Dyslipidemia	180 (50.7)	115 (27.6)	36 (33.3)	<0.001	<0.001	0.002	0.239
Fibroscan							
CAP (dB/m)	313 (263, 344)	296 (250, 332)	267 (231, 306)	<0.001	0.003	<0.001	<0.001
E (kPa)	6.3 (4.9, 8.1)	5.3 (4.3, 6.9)	5.3 (4.2, 6.4)	<0.001	<0.001	0.001	0.953
Steatosis				<0.001	<0.001	<0.001	<0.001
Grade 0	99 (27.9)	147 (35.3)	55 (50.9)				
Grade 1	26 (7.3)	32 (7.7)	17 (15.7)				
Grade 2	52 (14.7)	95 (22.8)	16 (14.8)				
Grade 3	178 (50.1)	143 (34.3)	20 (18.5)				
Fibrosis				<0.001	<0.001	<0.001	0.179
Stage 0	200 (56.34)	302 (72.4)	84 (77.8)				
Stage 1	43 (12.11)	40 (9.6)	9 (8.3)				
Stage 2	60 (16.90)	46 (11.0)	10 (9.3)				
Stage 3	37 (10.42)	18 (4.3)	4 (3.7)				
Stage 4	15 (4.23)	11 (2.6)	1 (0.9)				

Values are presented as the median (interquartile range) or frequency (percentage)

BMI: body mass index; WC: waist circumference; PBF: percent body fat; VFA: visceral fat area; ASM: appendicular skeletal muscle mass; ASMI: appendicular skeletal muscle mass index; FM: fat mass; FFM: fat free mass; FFM/FM: fat free mass to fat mass ratio; ALT: alanine aminotransferase; AST: aspartate aminotransferase; AKP: alkaline phosphatase; GGT: γ­glutamyl transferase; FBG: fasting blood glucose; FINS: fasting insulin; HOMA-IR: homeostasis model assessment of insulin resistance; TG: triglyceride; TC: total cholesterol; HDL: high-density lipoprotein; LDL: low-density lipoprotein; T2DM: type 2 diabetes mellitus; CAP: controlled attenuation parameter; E: elasticity

(a) P < 0.05 was considered statistically significant; (b) For multiple testing of post hoc analyses, p < 0.0167 (0.05/3) was considered statistically significant

**Fig. 3:**
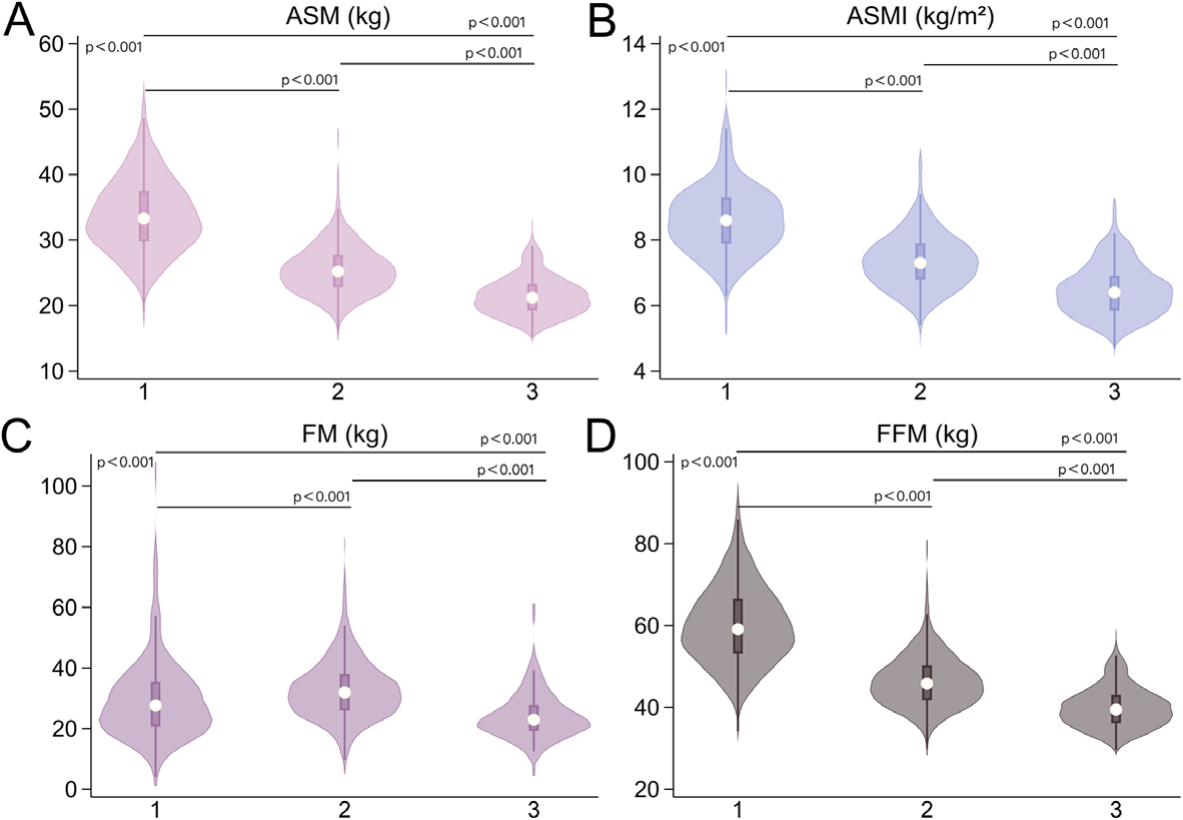
Comparison of ASM, ASMI, FM, and FFM values between the three groups. 1. Men; 2. Pre-menopausal women; 3. Post-menopausal women; ASM: appendicular skeletal muscle mass; ASMI: appendicular skeletal muscle mass index; FM: fat mass; FFM: fat free mass

**Fig. 4:**
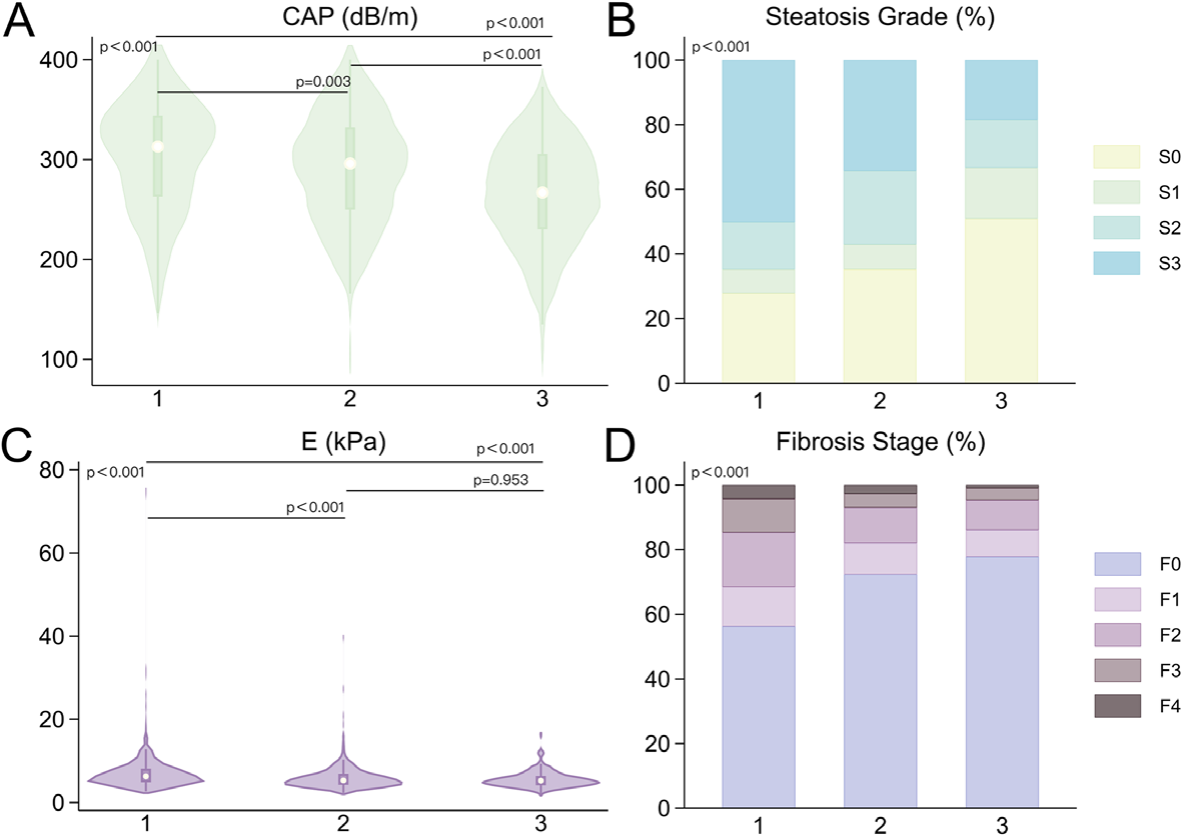
Comparison of liver steatosis and fibrosis and CAP/LSM-based grade distribution between the three groups. 1. Men; 2. Pre-menopausal women; 3. Post-menopausal women; CAP: controlled attenuation parameter; LSM: liver stiffness measurement; E: elasticity

### Relationship of liver steatosis and fibrosis severity to body composition variables

**Fig. 5:**
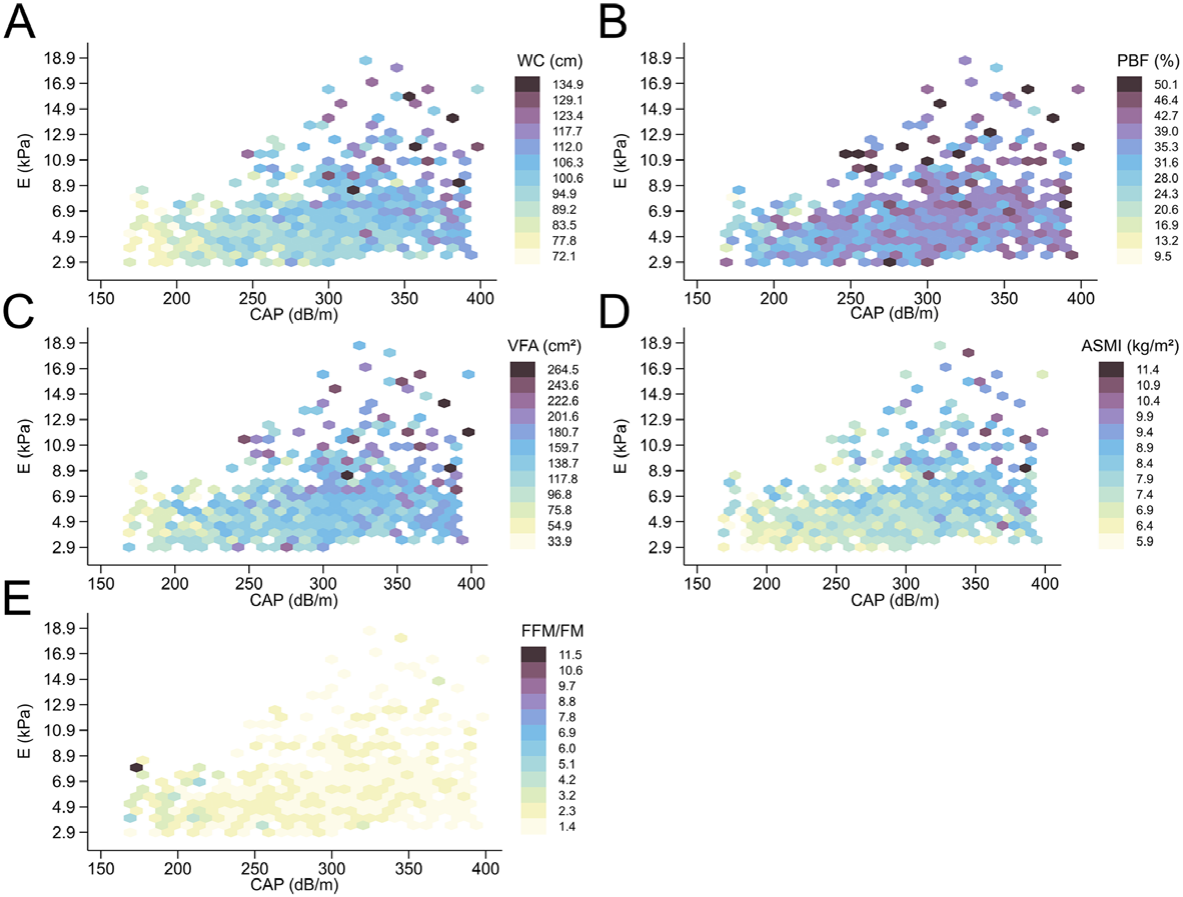
Relationship between body parameters (WC, PBF, VFA, ASMI, FFM/FM), CAP, and LSM values. WC: waist circumference; PBF: percent body fat; VFA: visceral fat area; ASMI: appendicular skeletal muscle mass index; FFM/FM: fat free mass to fat mass ratio; CAP: controlled attenuation parameter; LSM: liver stiffness measurement; E: elasticity

Heat plots were drawn to visualize the relationship between body parameters, CAP, and LSM values. The heat plots show that, in general, as liver steatosis and fibrosis progress, WC, PBF, VFA, and ASMI values gradually increase, and FFM/FM values gradually decrease (Fig. 5A-E).

### Interactions of body composition and sex or menopausal status on liver steatosis and fibrosis

For liver steatosis, there was an interaction between WC, visceral obesity, ASMI, and FFM/FM with sex (P = 0.005; P = 0.018; P = 0.009; P = 0.033, respectively); whereas for the interaction between body composition and menopausal status, it was observed only in visceral obesity (P = 0.033). For liver fibrosis, there was no significant interaction between body composition and sex or menopausal status (Additional Table S3). To understand the directionality of the interactions and effect sizes, we conducted logistic regression analyses and stratified by sex and menopausal status.

Univariate analysis showed that regardless of sex, menopausal status, WC, visceral obesity, ASMI, and FFM/FM were all significantly associated with liver steatosis grade (P < 0.001) (Table 2). For liver fibrosis, WC, ASMI, and FFM/FM were significantly associated with liver fibrosis stage in all three groups (P < 0.001), whereas the association between visceral obesity and liver fibrosis stage was significant in men and pre-menopausal women (P < 0.001) but not in post-menopausal women (P = 0.133) (Table 3).

**Table 2:** Univariate and multivariate analyses for liver steatosis severity in different sex and menopausal status populations

	Unadjusted		Model 1		Model 2		Model 3	
	OR (95% CI)	P value	OR (95% CI)	P value	OR (95% CI)	P value	OR (95% CI)	P value
Men								
WC (cm)	1.12 (1.09, 1.13)	<0.001	1.09 (1.05, 1.15)	< 0.001	1.07 (1.02, 1.13)	0.008	1.07 (1.02, 1.12)	0.011
VFA ≥ 100 cm²	7.22 (4.6, 11.31)	<0.001	1.62 (0.88, 2.98)	0.121	1.31 (0.70, 2.45)	0.395	1.33 (0.70, 2.49)	0.382
ASMI (kg/m²)	3.05 (2.35, 3.95)	<0.001	0.83 (0.51, 1.35)	0.461	0.87 (0.53, 1.42)	0.576	0.85 (0.51, 1.40)	0.525
FFM/FM	0.19 (0.13, 0.28)	<0.001	0.50 (0.31, 0.83)	0.007	0.55 (0.33, 0.92)	0.023	0.52 (0.31, 0.89)	0.017
Pre-menopausal women								
WC (cm)	1.07 (1.045, 1.09)	<0.001	1.07 (1.03, 1.11)	0.001	1.04 (0.998, 1.08)	0.059	1.04 (0.996, 1.08)	0.073
VFA ≥ 100 cm²	4.00 (2.02, 7.94)	<0.001	1.62 (0.76, 3.47)	0.210	1.47 (0.67, 3.21)	0.339	1.62 (0.74, 3.58)	0.228
ASMI (kg/m²)	1.96 (1.56, 2.47)	<0.001	1.08 (0.74, 1.56)	0.690	0.94 (0.64, 1.39)	0.763	0.95 (0.64, 1.41)	0.798
FFM/FM	0.28 (0.17, 0.46)	<0.001	0.83 (0.44, 1.55)	0.552	0.72 (0.36, 1.43)	0.345	0.67 (0.34, 1.35)	0.265
Post-menopausal women								
WC (cm)	1.08 (1.04, 1.13)	<0.001	1.08 (0.98, 1.19)	0.103	1.09 (0.99, 1.20)	0.094	1.10 (0.99, 1.21)	0.076
VFA ≥ 100 cm²	5.97 (2.07, 17.21)	0.001	3.78 (1.13, 12.65)	0.031	3.89 (1.05, 14.36)	0.041	4.16 (1.09, 15.90)	0.037
ASMI (kg/m²)	1.94 (1.23, 3.07)	0.005	0.74 (0.31, 1.75)	0.495	0.76 (0.32, 1.84)	0.549	0.94 (0.38, 2.30)	0.884
FFM/FM	0.13 (0.05, 0.39)	<0.001	0.22 (0.05, 1.01)	0.051	0.39 (0.09, 1.75)	0.219	0.42 (0.10, 1.75)	0.233

Multivariate model 1, adjusted for age and body mass index (BMI)

Multivariate model 2, adjusted for liver enzymes and dyslipidemia based on model 1

Multivariate model 3, adjusted for type 2 diabetes mellitus (T2DM) and homeostasis model assessment of insulin resistance (HOMA-IR) based on model 2

OR: odds ratio; CI: confidence interval; WC: waist circumference; VFA: visceral fat area; ASMI: appendicular skeletal muscle mass index; FFM/FM: fat free mass to fat mass ratio

**Table 3:** Univariate and multivariate analyses for liver fibrosis severity in different sex and menopausal status populations

	Unadjusted		Model 1		Model 2		Model 3	
	OR (95% CI)	P value	OR (95% CI)	P value	OR (95% CI)	P value	OR (95% CI)	P value
Men								
WC (cm)	1.07 (1.05, 1.08)	<0.001	1.06 (1.03, 1.10)	< 0.001	1.04 (1.01, 1.08)	0.017	1.05 (1.01, 1.09)	0.013
VFA ≥ 100 cm²	7.81 (4.47, 13.64)	<0.001	3.92 (2.05, 7.51)	< 0.001	3.59 (1.82,7.07)	< 0.001	3.92 (1.97, 7.81)	< 0.001
ASMI (kg/m²)	2.16 (1.74, 2.69)	<0.001	0.99 (0.64, 1.53)	0.954	0.92 (0.58, 1.44)	0.709	0.89 (0.56, 1.39)	0.598
FFM/FM	0.32 (0.22, 0.45)	<0.001	0.76 (0.47, 1.22)	0.262	0.85 (0.54, 1.33)	0.470	0.79 (0.49, 1.29)	0.351
Pre-menopausal women								
WC (cm)	1.07 (1.05, 1.10)	<0.001	1.03 (0.98, 1.08)	0.209	1.00 (0.95, 1.05)	0.943	1.01 (0.96, 1.06)	0.828
VFA ≥ 100 cm²	3.02 (1.16, 7.87)	0.024	0.87 (0.31, 2.48)	0.798	1.10 (0.33, 3.62)	0.874	1.16 (0.35, 3.84)	0.804
ASMI (kg/m²)	2.11 (1.62, 2.75)	<0.001	0.84 (0.53, 1.33)	0.449	0.72 (0.44, 1.17)	0.185	0.75 (0.45, 1.23)	0.250
FFM/FM	0.15 (0.07, 0.32)	<0.001	0.78 (0.31, 1.96)	0.600	0.58 (0.21, 1.61)	0.299	0.60 (0.22, 1.66)	0.322
Post-menopausal women								
WC (cm)	1.05 (1.00, 1.10)	0.036	0.93 (0.83, 1.04)	0.199	0.93 (0.82, 1.05)	0.229	0.93 (0.82, 1.05)	0.237
VFA ≥ 100 cm²	2.70 (0.74, 9.89)	0.133	0.98 (0.22, 4.46)	0.981	0.73 (0.14, 3.77)	0.706	0.71 (0.14, 3.69)	0.686
ASMI (kg/m²)	1.79 (1.02, 3.16)	0.044	0.82 (0.26, 2.64)	0.745	0.98 (0.32, 3.03)	0.970	1.01 (0.31, 3.22)	0.993
FFM/FM	0.20 (0.05, 0.82)	0.025	0.74 (0.15, 3.59)	0.713	0.86 (0.28, 2.60)	0.786	0.89 (0.32, 2.43)	0.815

Multivariate model 1, adjusted for age and body mass index (BMI)

Multivariate model 2, adjusted for liver enzymes and dyslipidemia based on model 1

Multivariate model 3, adjusted for type 2 diabetes mellitus (T2DM) and homeostasis model assessment of insulin resistance (HOMA-IR) based on model 2

OR: odds ratio; CI: confidence interval; WC: waist circumference; VFA: visceral fat area; ASMI: appendicular skeletal muscle mass index; FFM/FM: fat free mass to fat mass ratio

### Increased WC and low FFM/FM in men and visceral obesity in post-menopausal women are independently associated with more severe liver steatosis

Although some body composition data were significantly associated with liver steatosis in univariate analyses, other data such as age, BMI, and laboratory data differed between the three groups. To exclude the influence of these factors, we performed MOLR analyses to identify independent correlates of liver steatosis grade. As shown in Table 2, after adjusting for age and BMI, male WC (OR, 1.09; 95% CI, 1.05–1.15; P < 0.001) and FFM/FM (OR, 0.50; 95% CI, 0.31–0.83; P = 0.007), pre-menopausal female WC (OR, 1.07; 95% CI, 1.03–1.11; P = 0.001), post-menopausal female visceral obesity (OR, 3.78; 95% CI, 1.13–12.65; P = 0.031) were significantly related to liver steatosis grade (model 1). However, after controlling for liver enzymes and dyslipidemia, the correlation between WC and liver steatosis in pre-menopausal women became non-significant (model 2; OR, 1.04; 95% CI, 0.998–1.08, P = 0.059). Finally, after further adjustment for HOMA-IR and T2DM, WC (OR, 1.07; 95% CI, 1.02–1.12; P = 0.011) and FFM/FM (OR, 0.52; 95% CI, 0.31–0.89; P = 0.017) in men and visceral obesity (OR, 4.16; 95% CI, 1.09–15.90; P = 0.037) in post-menopausal women were still significantly associated with liver steatosis grade (model 3). Besides, after several model corrections, the correlation between ASMI and liver steatosis grade was no longer significant, irrespective of sex and menopausal status.

### Increased WC, and visceral obesity are independently associated with worse liver fibrosis in men

Similarly, we explored independent correlates of liver fibrosis stage across sex and menopausal status. As seen in Table 3, after adjusting for age and BMI, only WC (OR, 1.06; 1.03–1.10; P < 0.001), and visceral obesity (OR, 3.92; 95% CI, 2.05–7.51; P < 0.001) were significantly associated with liver fibrosis stage in men. These correlations did not change significantly after sequentially correcting for liver enzymes and dyslipidemia (model 2; OR, 1.04; 95% CI, 1.01–1.08; P = 0.017; OR, 3.59; 95% CI, 1.82–7.07; P < 0.001, respectively), HOMA-IR and T2DM (model 3; OR, 1.05; 95% CI, 1.01–1.09, P = 0.013; OR, 3.92; 95% CI, 1.97–7.81; P < 0.001, respectively). Besides, after correcting for multiple confounders, ASMI and FFM/FM in men and WC, visceral obesity, ASMI, and FFM/FM in pre- or post-menopausal women were not significantly associated with liver fibrosis stage.

## Discussion

Currently, most published clinical studies of NAFLD have failed to adequately analyze the impact of sex differences, particularly in terms of reproductive status. Our findings suggested that increased WC and low FFM/FM in men and visceral obesity in post-menopausal women are independently associated with more severe liver steatosis. Furthermore, increased WC and visceral obesity are independently associated with worse liver fibrosis in men.

Body composition differs by sex, with men and women having very different distributions and contents of muscle and fat. In general, women have less muscle and higher body fat compared to men [6]. Moreover, women tend to store fat in the subcutaneous and femoral regions, while men tend to store fat in the abdominal and visceral regions [6, 21]. Interestingly, estrogen influences the distribution of adipose tissue throughout a woman’s life. The decline of estrogen after menopause causes fat deposition to shift to the visceral region [22]. Except for VFA, the results of our study show that other body data are largely consistent with the above: Women showed more body fat, more FM, lower WC, lower ASM, and lower FFM than men. However, VFA did not match the above results. VFA was higher in pre-menopausal women than in men, as well as in post-menopausal women. The difference in VFA in pre- and post-menopausal women may stem from a significant BMI difference between the two groups in this study (30.1 vs. 25.4, P < 0.001), resulting in lower VFA in post-menopausal women. However, given the similar BMI of men and pre-menopausal women, such an observation is quite surprising. The mechanisms underlying sex differences in fat distribution are multifactorial and complex and may include sex hormones, cell-intrinsic factors, fat depot microenvironments, and tissue-specific genetic variation [21]. Strikingly, with the development of human genetics, a high percentage of sex-differentiated loci have been found in the genetics of fat distribution, providing new insights into sex differences in central obesity [21]. Multiple studies have shown that more than 50% of the central obesity loci have significant but poorly understood sexual dimorphism, with most loci having a stronger effect in females, which may explain the inversion of VFA between the sexes in our study [23–25].

Sex differences also exist in NAFLD, with epidemiological studies revealing that men have a higher prevalence and severity of NAFLD than women [4, 26]. Our results are similar, with greater CAP and LSM values in men, implying more severe liver steatosis and fibrosis than in pre- or post-menopausal women. The lower degree of steatosis in women compared to men seems puzzling considering the increased fat mass and decreased skeletal muscle mass in women. There are three possible reasons: First, women have more lipolytic activity in adipose tissue than men and rely more on free fatty acids (FFA) for energy. Simultaneously, they are more efficient in handling FFA and thus retain their insulin sensitivity [6]. In addition, one study found that obese men are less sensitive to insulin than obese women [27]. Second, female adipose tissue secretes more leptin and adiponectin, both of which are important adipokines that regulate metabolism and increase insulin sensitivity, respectively [28, 29]. Third, estrogen protects against liver steatosis. In mouse models, estrogen was found to have a protective effect against IR by activating estrogen receptor (ER) α in insulin-sensitive tissues [30]. Activation of the ERα signaling pathway in hepatocytes increased insulin sensitivity and limited hepatic fat deposition in female mice with a high-fat diet [31]. In contrast, disturbed hepatic lipid metabolism in ERα-deficient mice causes liver steatosis and exacerbates endoplasmic reticulum stress and inflammation [32]. In addition, as mentioned earlier, altered fat distribution in post-menopausal women causes a series of changes such as IR and increased incidence of metabolic syndrome (MetS) and leads to an increased risk of NAFLD in post-menopausal women [22, 33]. However, in our study, both CAP and the proportion of severe steatosis were lower in post-menopausal women than in pre-menopausal women, which may be due to a significant difference in BMI between the two populations. For liver fibrosis, many animal studies support the hypothesis that estrogen inhibits liver fibrosis by activating ERβ and inhibiting the activation and proliferation of hepatic stellate cells [34, 35]. A study regarding the effects of human sex and menopause on the severity of fibrosis also supports the protective effect of estrogen on fibrogenesis [36]. Notably, there were no significant differences in LSM values and liver fibrosis stage distribution between pre-menopausal and post-menopausal women, suggesting that estrogen may not be the only sex-specific factor affecting liver fibrosis. The sexual dimorphism of the liver histology in mice suggests that the human liver may also hide a sexual dimorphism. Normal livers of male rats tend to be more collagen-rich compared to those of female rats, whereas female rats have less fibrotic tissue, more Kupffer cells, and higher hepatocellularity [37].

A number of studies have also reported physical data associated with NAFLD, such as visceral obesity [7], increased WC and PBF [38], as risk factors for NAFLD and progression to liver fibrosis. From our heat plots, the trend of WC, PBF, and VFA as NAFLD progression is the same as the results of the above studies. Such results are not surprising considering the link between obesity and NAFLD. In particular, visceral fat, although accounting for only 7–15% of total body fat, is a major source of FFA for the liver, contributing to the development of inflammation and IR and driving the onset and progression of NASH [39, 40]. Of these physical indicators, the relationship between ASMI and NAFLD deserves further investigation. Sarcopenia, a disorder of low skeletal muscle mass, is thought to be associated with severe liver steatosis and fibrosis [8, 9]. However, our findings showed that ASMI increases with the severity of NAFLD. The discrepancy may come from different definitions of ASMI for diagnosing sarcopenia. A study found that severe liver steatosis was associated with an increased risk of sarcopenia as defined by the weight-adjusted ASMI (OR 1.73; 95% CI 1.31–2.28). In contrast, when ASMI was adjusted for height, the definition used in our study, severe liver steatosis was associated with a decreased risk of sarcopenia (OR, 0.63; 95% CI, 0.46–0.87) [41]. Such a result seems reasonable, as with weight gain comes a small increase in muscle mass to maintain daily physical activity [42], but height is relatively constant. For obese patients, this means a lower muscle mass index adjusted for weight and a higher muscle mass index adjusted for height. FFM/FM can be thought of as the muscle to fat ratio. This indicator is related to “sarcopenic obesity”, which is characterized by the presence of both sarcopenia and obesity [43]. FFM/FM has been found to be negatively associated with IR, MetS, and liver fat accumulation [44, 45]. This indicator also tends to decrease with increasing liver steatosis and fibrosis on our heat plots, corresponding to lower muscle mass and higher fat mass. Muscle loss leads to IR, while excess adipose tissue causes increased FFA and chronic inflammation, and its interaction with sarcopenia exacerbates muscle loss. These factors act on the liver, causing NAFLD and accelerating liver fibrosis [46].

After exploring the two-by-two relationships between NAFLD, sex and reproductive status, and body composition, we wonder about the interaction between the three. Does the relationship between body composition and NAFLD differ by sex or reproductive status? Therefore, we performed interaction analyses, which showed that for liver steatosis, there was an interaction between sex and WC, or visceral obesity, or ASMI, or FFM/FM, and an interaction between menopause and visceral obesity. No significant interaction of body composition with sex or menopausal status was found in liver fibrosis. We further performed MOLR analyses stratified by sex and menopause and controlled for confounders to explore independent correlates of liver steatosis and fibrosis. We found that WC and FFM/FM were independently associated with liver steatosis grade in men, whereas visceral obesity was only found to be independently associated with liver steatosis grade in post-menopausal women. Women’s thicker subcutaneous fat may interfere with WC, preventing it from accurately reflecting abdominal and liver fat. A study also showed that WC was only associated with liver fat accumulation in men but not in women [10]. One study, using FM/FFM, found a higher risk ratio for NAFLD in men than in women with higher FM/FFM (1.55 vs. 1.42 for non-obese; 1.33 vs. 1.29 for obese) [47]. This also suggests that the inherent differences in fat metabolism, insulin sensitivity, and sex hormones between men and women make sarcopenic obesity more harmful for men. Notably, visceral obesity was only independently associated with liver steatosis grade in post-menopausal women. This may be driven by a shift of fat to the viscera in women after menopause, which makes visceral obesity more susceptible to fatty liver than in pre-menopausal women and men. At the same time, such findings fully illustrate the influence of female menopause on liver histology in addition to sex. In the analysis of independent correlates of liver fibrosis, only WC,and visceral obesity were found to be independently associated with liver fibrosis stage in men. Increased WC, and VFA both reflect fat accumulation with subsequent inflammation and IR. On this basis, differences in liver fibrotic tissue, adipose muscle metabolism, and estrogen between the sexes contribute to the male susceptibility to liver fibrosis and drive fibrosis progression.

Our study has several strengths: Firstly, most studies have analyzed sex as a variable, and there is a lack of studies stratified by sex and menopausal status. We examined the association of body composition with liver steatosis and fibrosis across sex and reproductive status. Secondly, we assessed body composition indicators more comprehensively. Finally, the study included a relatively large sample size, including 880 patients. However, we should also acknowledge these limitations: Firstly, body composition was measured by BIA rather than computed tomography, magnetic resonance imaging, or dual-energy x-ray absorptiometry. Although these techniques are considered more reliable, they are limited by cost and difficult to apply on a large scale. Currently, AWGS also supports the use of multi-frequency BIA [14]. Secondly, we did not use liver biopsy to evaluate liver histology. Thirdly, the small sample size of post-menopausal women affects the efficacy of our analysis. Fourthly, the lack of a longitudinal design in the study limited our ability to establish a causal relationship between body composition and the severity of liver steatosis and fibrosis in NAFLD. Finally, our study sample was based on Chinese hospital patients, a significant proportion of whom had comorbid T2DM or dyslipidemia. Therefore, the results need to be validated in a more ethnically diverse, generalized population.

## Conclusions

Our study supported the existence of differences in body composition across sex and reproductive status that differentially affect their respective NAFLD. Increased WC and low FFM/FM in men and visceral obesity in post-menopausal women were independent correlates of more severe liver steatosis. Besides, increased WCand visceral obesity were independent correlates of worse liver fibrosis in men. Therefore, we emphasized the sex- and reproductive status-specific management of NAFLD. WC has added value as a more routine and simple measurement for the identification of NAFLD in men, and fat loss and muscle gain are important strategies for the management of NAFLD in men. For post-menopausal women, the important thing is to reduce visceral fat. In the future, more consideration of sex and reproductive status specificity, as well as longitudinally designed clinical studies, will be needed to achieve accurate management of NAFLD.

### Electronic supplementary material

Below is the link to the electronic supplementary material.

Supplementary Material 1

## Data Availability

The datasets used and/or analyzed during the current study are available from the corresponding author on reasonable request.

## Abbreviations

NAFLD: Non-alcoholic fatty liver disease
NASH: Nonalcoholic steatohepatitis
BMI: Body mass index
VFA: Visceral fat area
ASM: Appendicular skeletal muscle mass
WC: Waist circumference
LSM: Liver stiffness measurement
CAP: Controlled attenuation parameter
IQR: Interquartile range
BIA: Bioelectrical-impedance body composition analyzer
PBF: Percent body fat
FM: Fat mass
FFM: Fat free mass
ASMI: Appendicular skeletal muscle mass index
AWGS: Asian Working Group for Sarcopenia
ALT: Alanine aminotransferase
AST: Aspartate aminotransferase
GGT: γ­glutamyl transferase
AKP: Alkaline phosphatase
FBG: Fasting blood glucose
FINS: Fasting insulin
HOMA-IR: Homeostasis model assessment of insulin resistance
TC: Total cholesterol
TG: Triglyceride
HDL: High-density lipoprotein
LDL: Low-density lipoprotein
IR: Insulin resistance
T2DM: Type 2 diabetes mellitus
HbA1c: Hemoglobin A1c
ANOVA: Analysis of variance
K-H: Kruskal-Wallis
MOLR: Multiple ordinal logistic regression
OR: Odds ratio
CI: Confidence interval
FFA: Free fatty acids
ER: Estrogen receptor
MetS: Metabolic syndrome
