# Supplementary material for: Sex- and reproductive status-specific relationships between body composition and non-alcoholic fatty liver disease
Source: BMC Gastroenterol. 2023 Oct 24;23:364. doi: 10.1186/s12876-023-02997-9 (PMC10598923; doi:10.1186/s12876-023-02997-9)
Supplement: Supplementary file 1 — Supplementary Material 1 [file 12876_2023_2997_MOESM1_ESM.docx]

Table S1. The Strengthening the Reporting of Observational Studies in Epidemiology Statement.

| **Section/Topic** | **Item No** | **Recommendation** | **Reported on**  Page/Section/Paragraph(s) |
| --- | --- | --- | --- |
| **Title and Abstract** | 1 | (*a*) Indicate the study’s design with a commonly used term in the title or the abstract | Abstract |
|  |  | (*b*) Provide in the abstract an informative and balanced summary of what was done and what was found | Abstract |
| **Introduction** | | | |
| Background/  rationale | 2 | Explain the scientific background and rationale for the investigation being reported | Introduction, Paragraph 1, 2 |
| Objectives | 3 | State specific objectives, including any prespecified hypotheses | Introduction, Paragraph 3 |
| **Methods** | | | |
| Study design | 4 | Present key elements of study design early in the paper | Patients and methods, Paragraph 1 |
| Setting | 5 | Describe the setting, locations, and relevant dates, including periods of recruitment, exposure, follow-up, and data collection | Patients and methods, Paragraph 2 |
| Participants | 6 | (*a*) *Cohort study*—Give the eligibility criteria, and the sources and methods of selection of participants. Describe methods of follow-up  *Case-control study*—Give the eligibility criteria, and the sources and methods of case ascertainment and control selection. Give the rationale for the choice of cases and controls  *Cross-sectional study*—Give the eligibility criteria, and the sources and methods of selection of participants | Patients and methods, Paragraph 2 |
|  |  | (*b*) *Cohort study*—For matched studies, give matching criteria and number of exposed and unexposed  *Case-control study*—For matched studies, give matching criteria and the number of controls per case | NA |
| Variables | 7 | Clearly define all outcomes, exposures, predictors, potential confounders, and effect modifiers. Give diagnostic criteria, if applicable | NA |
| Data sources/ measurement | 8* | For each variable of interest, give sources of data and details of methods of assessment (measurement). Describe comparability of assessment methods if there is more than one group | Patients and methods, Paragraph 4-8 |
| Bias | 9 | Describe any efforts to address potential sources of bias | NA |
| Study size | 10 | Explain how the study size was arrived at | NA |
| Quantitative variables | 11 | Explain how quantitative variables were handled in the analyses. If applicable, describe which groupings were chosen and why | NA |
| Statistical methods | 12 | (*a*) Describe all statistical methods, including those used to control for confounding | Patients and methods, Paragraph 9-10 |
|  |  | (*b*) Describe any methods used to examine subgroups and interactions | Patients and methods, Paragraph 10 |
|  |  | (*c*) Explain how missing data were addressed | NA |
|  |  | (*d*) *Cohort study*—If applicable, explain how loss to follow-up was addressed  *Case-control study*—If applicable, explain how matching of cases and controls was addressed  *Cross-sectional study*—If applicable, describe analytical methods taking account of sampling strategy | NA |
|  |  | (*e*) Describe any sensitivity analyses | Patients and methods, Paragraph 10 |
| **Results** | | | |
| Participants | 13* | (a) Report numbers of individuals at each stage of study—*e.g.*, numbers potentially eligible, examined for eligibility, confirmed eligible, included in the study, completing follow-up, and analysed | Figure 1 |
|  |  | (b) Give reasons for non-participation at each stage | Figure 1 |
|  |  | (c) Consider use of a flow diagram | Figure 1 |
| Descriptive data | 14* | (a) Give characteristics of study participants (*e.g.*, demographic, clinical, social) and information on exposures and potential confounders | Table 1 |
|  |  | (b) Indicate number of participants with missing data for each variable of interest | NA |
|  |  | (c) *Cohort study*—Summarise follow-up time (*e.g.*, average and total amount) | NA |
| Outcome data | 15* | *Cohort study*—Report numbers of outcome events or summary measures over time | NA |
|  |  | *Case-control study—*Report numbers in each exposure category, or summary measures of exposure | NA |
|  |  | *Cross-sectional study—*Report numbers of outcome events or summary measures | Table 1 |
| Main results | 16 | (*a*) Give unadjusted estimates and, if applicable, confounder-adjusted estimates and their precision (*e.g.*, 95% confidence interval). Make clear which confounders were adjusted for and why they were included | Table 2 and 3 |
|  |  | (*b*) Report category boundaries when continuous variables were categorized | Patients and methods, Paragraph 5 and 6 |
|  |  | (*c*) If relevant, consider translating estimates of relative risk into absolute risk for a meaningful time period | NA |
| Other analyses | 17 | Report other analyses done—*e.g.*, analyses of subgroups and interactions, and sensitivity analyses | Table 2 and 3 |
| **Discussion** | | | |
| Key results | 18 | Summarise key results with reference to study objectives | Discussion, Paragraph 1 |
| Limitations | 19 | Discuss limitations of the study, taking into account sources of potential bias or imprecision. Discuss both direction and magnitude of any potential bias | Discussion, Paragraph 6 |
| Interpretation | 20 | Give a cautious overall interpretation of results considering objectives, limitations, multiplicity of analyses, results from similar studies, and other relevant evidence | Discussion, Paragraph 2-5 |
| Generalisability | 21 | Discuss the generalisability (external validity) of the study results | Conclusions |
| **Other Information** | | | |
| Funding | 22 | Give the source of funding and the role of the funders for the present study and, if applicable, for the original study on which the present article is based | Funding |

NA: Not applicable.

Table S2. The test power.

|  | Comparison between three groups | | Post-hoc | | | | | |
| --- | --- | --- | --- | --- | --- | --- | --- | --- |
|  |  |  | 1 vs 2 | | 1 vs 3 | | 2 vs 3 | |
|  | P value^a^ | Power | P value^b^ | Power | P value^b^ | Power | P value^b^ | Power |
| Age (year) | <0.001 | 1.0000 | <0.001 | 1.0000 | <0.001 | 1.0000 | <0.001 | 1.0000 |
| BMI (kg/m^2^) | <0.001 | 1.0000 | 1.000 | 0.0251 | <0.001 | 1.0000 | <0.001 | 1.0000 |
| WC (cm) | <0.001 | 1.0000 | 0.002 | 0.8091 | <0.001 | 1.0000 | <0.001 | 1.0000 |
| PBF (%) | <0.001 | 1.0000 | <0.001 | 1.0000 | <0.001 | 1.0000 | <0.001 | 0.9999 |
| VFA (cm^2^) | <0.001 | 1.0000 | <0.001 | 1.0000 | 1.000 | 0.0838 | <0.001 | 1.0000 |
| ASM (kg) | <0.001 | 1.0000 | <0.001 | 1.0000 | <0.001 | 1.0000 | <0.001 | 1.0000 |
| ASMI (kg/m²) | <0.001 | 1.0000 | <0.001 | 1.0000 | <0.001 | 1.0000 | <0.001 | 1.0000 |
| FM (kg) | <0.001 | 1.0000 | <0.001 | 0.9490 | <0.001 | 0.9982 | <0.001 | 1.0000 |
| FFM (kg) | <0.001 | 1.0000 | <0.001 | 1.0000 | <0.001 | 1.0000 | <0.001 | 1.0000 |
| FFM/FM | <0.001 | 1.0000 | <0.001 | 1.0000 | <0.001 | 1.0000 | 0.004 | 0.9672 |
| ALT (U/L) | <0.001 | 0.9999 | <0.001 | 0.9568 | <0.001 | 1.0000 | 0.024 | 0.9632 |
| AST (U/L) | 0.001 | 0.9153 | 0.016 | 0.6166 | 0.005 | 0.9983 | 0.516 | 0.3884 |
| AKP (U/L) | <0.001 | 1.0000 | <0.001 | 0.9917 | 0.101 | 0.3233 | <0.001 | 0.9937 |
| GGT (U/L) | <0.001 | 1.0000 | <0.001 | 1.0000 | <0.001 | 0.9647 | 1.000 | 0.0190 |
| FBG (mmol/L) | <0.001 | 1.0000 | <0.001 | 1.0000 | 0.365 | 0.1786 | <0.001 | 0.9999 |
| FINS (μIU/mL) | <0.001 | 0.9953 | 0.592 | 0.1266 | <0.001 | 0.9732 | <0.001 | 0.9992 |
| HOMA-IR | 0.062 | 0.5529 | NA | NA | NA | NA | NA | NA |
| TG | <0.001 | 1.0000 | <0.001 | 1.0000 | <0.001 | 0.9922 | 1.000 | 0.0770 |
| TC | 0.236 | 0.3119 | NA | NA | NA | NA | NA | NA |
| HDL | <0.001 | 1.0000 | <0.001 | 1.0000 | <0.001 | 1.0000 | <0.001 | 0.9494 |
| LDL | 0.172 | 0.3709 | NA | NA | NA | NA | NA | NA |
| T2DM | <0.001 | 1.0000 | <0.001 | 1.0000 | <0.001 | 0.8862 | <0.001 | 1.0000 |
| Dyslipidemia | <0.001 | 0.9923 | <0.001 | 1.0000 | 0.002 | 0.7812 | 0.239 | 0.1123 |
| CAP (dB/m) | <0.001 | 1.0000 | 0.003 | 0.8064 | <0.001 | 1.0000 | <0.001 | 0.9867 |
| E (kPa) | <0.001 | 0.9922 | <0.001 | 0.9144 | 0.001 | 0.9888 | 0.953 | 0.2169 |
| Steatosis | <0.001 | 0.9838 | <0.001 | 0.9046 | <0.001 | 0.9998 | <0.001 | 0.9305 |
| Fibrosis | <0.001 | 0.9570 | <0.001 | 0.9935 | <0.001 | 0.9603 | 0.179 | 0.1130 |

1. Men; 2. Pre-menopausal women; 3. Post-menopausal women; BMI: body mass index; WC: waist circumference; PBF: percent body fat; VFA: visceral fat area; ASM: appendicular skeletal muscle mass; ASMI: appendicular skeletal muscle mass index; FM: fat mass; FFM: fat free mass; FFM/FM: fat free mass to fat mass ratio; ALT: alanine aminotransferase; AST: aspartate aminotransferase; AKP: alkaline phosphatase; GGT: γ­glutamyl transferase; FBG: fasting blood glucose; FINS: fasting insulin; HOMA-IR: homeostasis model assessment of insulin resistance; TG: triglyceride; TC: total cholesterol; HDL: high-density lipoprotein; LDL: low-density lipoprotein; T2DM: type 2 diabetes mellitus; CAP: controlled attenuation parameter; E: elasticity; NA: Not applicable.

a. P < 0.05 was considered statistically significant; b. For multiple testing of post hoc analyses, p < 0.0167 (0.05/3) was considered statistically significant.

Table S3. Interactions of body composition and sex or menopausal status on liver steatosis and fibrosis.

| Interaction term | | Steatosis grade | Fibrosis stage |
| --- | --- | --- | --- |
|  |  | P value | P value |
| Sex | WC (cm) | 0.005 | 0.923 |
|  | VFA ≥ 100 cm² | 0.018 | 0.051 |
|  | ASMI (kg/m²) | 0.009 | 0.543 |
|  | FFM/FM | 0.033 | 0.083 |
| Menopausal status | WC (cm) | 0.677 | 0.350 |
|  | VFA ≥ 100 cm² | 0.033 | 0.539 |
|  | ASMI (kg/m²) | 0.855 | 0.589 |
|  | FFM/FM | 0.583 | 0.687 |

WC: waist circumference; VFA: visceral fat area; ASMI: appendicular skeletal muscle mass index; FFM/FM: fat free mass to fat mass ratio;
